# Clinical value of optical coherence tomography in the early diagnosis of cervical cancer and precancerous lesions: a cross-sectional study

**DOI:** 10.3389/fonc.2024.1423128

**Published:** 2024-07-29

**Authors:** Xiaoli Cui, Di Yang, Jing Zhang, Yuqian Zhao, Zhumei Cui, Chunyan Wang, Youlin Qiao

**Affiliations:** ^1^ Department of Gynecologic Oncology, Cancer Hospital of China Medical University, Liaoning Cancer Hospital & Institute, Shenyang, Liaoning, China; ^2^ Sichuan Cancer Hospital & Institute, Sichuan Cancer Center, School of Medicine, University of Electronic Science & Technology of China, Chengdu, Sichuan, China; ^3^ The Affiliated Hospital of Qingdao University, Qingdao, Shandong, China; ^4^ Chinese Academy of Medical Sciences & Peking Union Medical College, Beijing, China

**Keywords:** cervical cancer, precancerous lesions, OCT, early diagnosis, screening

## Abstract

**Background:**

This study aimed to measure the accuracy of optical coherence tomography (OCT) in the early diagnosis of high-grade cervical lesions and assess its diagnostic value in the triage of high-risk HPV infection.

**Method:**

From Jan 2019 to Jan 2021, women who visited the gynecology clinics of 2 hospitals for colposcopy were invited to participate in this study. Women aged 35 to 64 years old who were sexually active and had an intact cervix with a diameter of more than or equal to 2 cm were included in this study. Additionally, individuals with abnormal cytology, positive HPV test results, or other clinically suspicious symptoms or signs were referred. All participants were examined before colposcopy using OCT. Biopsy and/or ECC were conducted under colposcopy. We used the results of histopathology as the gold standard and assessed the accuracy of OCT.

**Results:**

Overall, 883 women were included in the analysis. Approximately 13.25% of women were ASCUS+ in cytological assessments, and 22.31% were positive for high-risk HPV. Nearly 15.18% of women were positive in OCT. Of them, 27 women were diagnosed with CIN2, and 33 were diagnosed with CIN3+ lesions. Among HPV-positive women, the detection rates for CIN2+ and CIN3+ were much lower for those who were negative in OCT, compared with NILM cytology (CIN2+: 20.0% vs. 30.0%, *P*=0.002, and CIN3+: 18.2% vs. 27.3%, *P*=0.013). Among women who were positive for HPV16/18, the detection rate for CIN2+ was much lower for negative OCT, compared with NILM cytology (8.3% vs.15.0%, *P*=0.005). Compared to HPV and cytological tests, HPV combined with OCT had higher specificity for detecting CIN2+ and CIN3+ (96.1% vs. 93.2%, *P*=0.002; 93.8% vs. 91.3%, *P*=0.013). OCT triage after HPV genotyping had the highest AUC for detecting CIN2+ and CIN3+ cases among patients with high-risk HPV infection (0.921, 0.920).

**Conclusion:**

OCT is an accurate test for the early diagnosis of high-grade cervical lesions and has great diagnostic value in the triage of patients with high-risk HPV infection.

## Introduction

Cervical cancer is a common malignancy among women, with 604,000 new cases and 342,000 deaths worldwide in 2020 (1). The incidence of cervical cancer in China is relatively high. In 2018, there were 106,430 new cases and 47,739 deaths because of cervical cancer in China, accounting for 18.2% of new cases and 17.3% of deaths worldwide (2). According to the Annual Report of the National Cancer Center in 2020, cervical cancer is still the 6th leading common cancer among women. Although conventional cervical cytology is a commonly used screening method due to its high specificity, it necessitates professional training of cytologists and sufficient infrastructure, which makes it difficult to meet the needs of many resource-poor areas (3). The test for high-risk human papillomavirus (HPV) is highly sensitive and non-invasive (4). In July 2021, the WHO Guidelines for the Screening and Treatment of Precancerous Cervical Lesions (2nd edition) explicitly proposed the use of HPV testing as the primary screening method for cervical cancer (5). HPV infection does not represent the development of cervical lesions or even cervical cancer. However, an HPV positive result can easily evoke anxiety among women, or lead to over-medication, unnecessary physical and mental harm, and waste of medical resources. Therefore, it is necessary to explore appropriate screening techniques in combination with HPV testing or triage techniques for HPV-positive populations.

Optical coherence tomography (OCT) is a new imaging technology. Through the phenomenon of light interference, it can scan organs and tissues using harmless near-infrared light and generate cell-level three-dimensional images of tissues in a real-time manner, aiding the diagnosis of diseases (6, 7). Moreover, OCT immediately reveals whether the patient is suspicious for cervical intraepithelial lesions (CIN) or cancer. In addition, physicians can proficiently operate the device after a short period of training. Zhang et al. (8) found that OCT imaging systems exhibit high sensitivity and specificity in evaluating cervical lesions, characterized by non-invasiveness, real-time capability, and efficiency. Xiao et al. (9) compared different triage strategies (cytology, OCT, and HPV) in colposcopy examinations. They observed that compared to cytology-based triage, combined OCT and high-risk HPV triage provide similar immediate risk stratification for CIN3+ and reduce the number of colposcopy examinations required. However, few studies have measured the clinical value of OCT in the early diagnosis of cervical cancer and precancerous lesions in China.

Herein, we aimed to evaluate the accuracy of OCT as a primary screening technique for cervical cancer. Furthermore, we used OCT in combination with HPV testing or as a triage technique for HPV-positive populations.

## Materials and methods

### Study population

From Jan 2019 to Jan 2021, women who visited Liaoning Cancer Hospital and the First Affiliated Hospital of Qingdao University for cervical cancer screening and underwent colposcopy were invited to participate in this study. The inclusion criteria were as follows: 1) sexually active women aged 35-64 years in good health condition; 2) having intact cervix with a diameter of greater than or equal to 2 cm; 3) voluntarily signing the informed consent form; and 4) patients meeting the criteria for referral for colposcopy, including those with abnormal cytology, positive hr-HPV, or other clinically suspicious symptoms or signs. The exclusion criteria were as follows: 1) women with suspicious symptoms of pregnancy or those within 8 weeks after delivery; 2) patients with a clear history of cervical cancer and precancerous lesions; 3) physically or mentally unable to undergo an examination or to provide informed consent. The order of testing was as follows: cytology, HPV test, OCT, colposcopy, and biopsy.

This study was approved by the Ethics Committee of Liaoning Cancer Hospital (Approval number: 20200101). All participants signed an informed consent form before the start of this study.

### Cytological examination

This study followed the 2014 Cervical Cytology Bethesda Reporting System, and the lesions included negative for intraepithelial lesion or malignancy (NILM), atypical squamous cells of undetermined significance (ASC-US), low-grade squamous intraepithelial lesion (LSIL), high-grade squamous intraepithelial lesion (HSIL), atypical squamous cells cannot exclude HSIL (ASC-H), atypical glandular cells (AGC) and squamous cell carcinoma (SCC).

### HPV test

The Aptima test can detect 14 subtypes of HPV, including HPV 16, 18, 31, 33, 35, 39, 45, 51, 52, 56, 58, 59, 66, and 68. Additionally, it can specifically detect HPV 16, 18/45, and other high-risk types. The researchers at the participating hospitals performed the test and reported the results following the instructions.

### OCT

During OCT, the participants were in the lithotomy position. After placing the speculum, the physician cleaned the vagina and cervical mucus and placed a special probe with a disposable OCT protective sleeve in the vagina, examining in a 360°clockwise direction from point 1 of the cervix to point 12. In total, 12 images of each point per cervix were captured and related data were saved and uploaded. After interpreting OCT images, trained researchers immediately obtained the results of a 12-point diagnosis and reported it as negative (including normal, inflammatory changes or low-grade cervical lesions associated with HPV) or positive (including high-grade cervical lesions and invasive cervical cancer). The physicians and trained researchers conducting OCT were blinded to the results of cytology and HPV test.

### Colposcopy and biopsy

OCT and colposcopy were conducted on the same day. Colposcopists were trained and proficient in performing colposcopy. Colposcopy was negative for intraepithelial lesions or malignant neoplasms, LSIL, HSIL, and cancer. After finding a suspicious cervical lesion, a biopsy was performed on the lesion site. In the absence of suspicious cervical lesions, random biopsies were performed at 3, 6, 9, and 12 o ‘clock. Endocervical curettage (ECC) was performed when the squamous-column junction was not completely visible. Finally, systematic reporting of pathological findings in cervical intraepithelial lesions (CIN) was used as the gold standard.

### Statistical analysis

Statistical analyses were performed using SPSS20.0. CIN2+ and CIN3+ lesions were used as endpoints to determine the detection rate, sensitivity, specificity, positive predictive value (PPV), negative predictive value (NPV), and area under the curve (AUC). The χ2 test was used to compare the rates, and the McNemar test was used for paired comparison. *P* values less than 0.05 were considered statistically significant.

## Results

### Population characteristics

In total, 883 participants with complete HPV, cytology, and OCT results were included in this study. There were 278 cases (31.48%) between 35 and 44 years, 373 cases (42.24%) between 45 and 54 years, and 232 cases (26.27%) between 55 and 65 years. Cytology was reported as ASC-US and above in 13.25% of participants. The HPV-positive rate was 22.31%, and among HPV-positive cases, 33.5% were positive for HPV16/18. The positive rate of OCT was 15.18%. Histopathological examination showed that 731 participants were normal, 92 participants had CIN1 lesions, 27 participants had CIN2 lesions, and 33 participants had CIN3+ lesions. The age distribution and clinical diagnosis of women included in this study are shown in **Table 1**.

**Table 1:** Distribution of histological diagnostic findings stratified by age group, cytology, HPV testing, and OCT examination of the included women [number (%)].

	Histological diagnosis [N (%)]				Total
	Normal	CIN1	CIN2	CIN3	
Age					
<45	187 (67.27)	47 (16.91)	21 (7.55)	23 (8.27)	278 (31.48)
45-55	331 (88.74)	32 (8.58)	4 (1.07)	6 (1.61)	373 (42.24)
>55	213 (91.81)	13 (5.6)	2 (0.86)	4 (1.72)	232 (26.27)
Cytology					
NILM	699 (91.25)	52 (6.79)	7 (0.91)	8 (1.04)	766 (86.75)
ASC-US	19 (38.78)	21 (42.86)	4 (8.16)	5 (10.2)	49 (5.55)
LSIL	12 (33.33)	14 (38.89)	8 (22.22)	2 (5.56)	36 (4.08)
ASC-H/AGC	1 (6.25)	2 (12.5)	3 (18.75)	10 (62.5)	16 (1.81)
HSIL+(HSIL/SCC)	0 (0)	3 (18.75)	5 (31.25)	8 (50)	16 (1.81)
HPV testing					
Negative	657 (95.77)	26 (3.79)	2 (0.29)	1 (0.15)	686 (77.69)
16/18 Positive	17 (25.76)	23 (34.85)	8 (12.12)	18 (27.27)	66 (7.47)
Other high-risk positive	57 (43.51)	43 (32.82)	17 (12.98)	14 (10.69)	131 (14.84)
OCT examination					
Negative	702 (93.72)	37 (4.94)	4 (0.53)	6 (0.8)	749 (84.82)
Positive	29 (21.64)	55 (41.04)	23 (17.16)	27 (20.15)	134 (15.18)
Total	731 (82.79)	92 (10.42)	27 (3.06)	33 (3.74)	883 (100)

HPV, human papillomavirus; OCT, optical coherence tomography; CIN, cervical intraepithelial lesions; NILM, negative for intraepithelial lesion or malignancy; ASC-US, atypical squamous cells of undetermined significance; LSIL, low-grade squamous intraepithelial lesion; ASC-H, atypical squamous cells cannot exclude HSIL; AGC, atypical glandular cells; HSIL+, high-grade squamous intraepithelial lesion or worse; HSIL, high-grade squamous intraepithelial lesion; SCC, squamous cell carcinoma.

### Comparison of lesion detection rate and accuracy among women with different clinical results

Considering CIN2+ as the end-point for clinical observation, the detection rate of OCT-positive women was 83.3%, higher than that in the cytological assessment of ASC-US+ lesions (83.3% vs. 75.0%, *P*<0.332). When considering CIN3+ as the endpoint, the detection rate was 81.8% among OCT-positive women, which was higher than that in cytology with ASC-US+ (81.8% vs. 75.8%, *P*=0.727). In HPV-positive women, the detection rates of CIN2+ and CIN3+ lesions were significantly lower in OCT-negative women than in cytological NILM (20.0% vs. 30.0%, *P*=0.002 and 18.2% vs. 27.3%, *P*=0.013, respectively). Among women positive for HPV16/18, the detection rate of CIN2+ lesions was significantly lower in OCT-negative women than in cytological NILM women (8.3% vs. 15.0%, *P*=0.005).

The accuracy of HPV test, cytological examination, OCT screening alone, or combined screening for CIN2+ and CIN3+ are shown in **Table 2**. When using ASC-US+ as the cut-off value, the sensitivity, specificity, and AUC of HPV untyped combined with cytological screening for CIN2+ were 70.0% (56.6%, 80.8%), 93.2%, (91.2%, 94.8%), and 0.816 (0.746, 0.886), respectively. The sensitivity, specificity, and AUC for HPV untyped combined with OCT screening were 80.0% (67.3%, 88.8%), 96.1% (94.5%, 97.3%), and 0.881 (0.820, 0.931), respectively. For CIN3+, the sensitivity, specificity, and AUC of HPV untyped combined with cytological screening were 72.7% (54.2%, 86.1%), 91.3% (89.1%, 93.1%), and 0.820 (0.730, 0.910), respectively. In contrast, the sensitivity, specificity, and AUC of HPV untyped combined with OCT screening were 81.8% (63.9%, 92.4%), 93.8% (91.9%, 95.3%), and 0.878 (0.800, 0.955), respectively. In our study, the specificity of HPV untyped test combined with OCT for screening CIN2+ and CIN3+ was significantly higher than that of HPV untyped test combined with cytology (96.1% vs. 93.2%, *P*=0.002 and 93.8% vs. 91.3%, *P*=0.013).

**Table 2:** Accuracy of different screening techniques and their combinations.

	Sensitivity (95% CI)	Specificity (95% CI)	PPV (95% CI)	NPV (95% CI)	AUC (95% CI)
CIN2+					
Cytology	75.0 (61.9, 84.9)	91.3 (89.1, 93.0)	38.5 (29.8, 47.9)	98.0 (96.7, 98.9)	0.831 (0.766, 0.897)
HPV	95.0 (85.2, 98.7)	83.0 (80.2, 85.5)	28.9(22.8, 35.9)	99.6(98.6, 99.9)	0.890 (0.854, 0.926)
OCT	83.3 (71.0, 91.3)	89.8 (87.5, 91.7)	37.3 (29.2, 46.1)	98.7 (97.5, 99.3)	0.866 (0.810, 0.922)
HPV untyped + cytology	70.0 (56.6, 80.8)	93.2 (91.2, 94.8)	42.9 (33.0, 53.2)	97.7 (96.3, 98.6)	0.816 (0.746, 0.886)
HPV untyped +OCT	80.0 (67.3, 88.8)	96.1 (94.5, 97.3)	60.0 (48.4, 70.6)	98.5 (97.3, 99.2)	0.881 (0.820, 0.941)
HPV typing + cytology*	85.0 (72.9, 92.5)	90.4 (88.1, 92.3)	39.2 (30.9, 48.2)	98.8 (97.7, 99.4)	0.877 (0.823, 0.931)
HPV typing+OCT*	91.7 (80.9, 96.9)	92.6 (90.5, 94.2)	47.4 (38.1, 56.9)	99.3 (98.4, 99.8)	0.921 (0.880, 0.963)
CIN3+					
Cytology	75.8 (57.4, 88.3)	89.2 (86.8, 91.1)	21.4 (14.6, 30.1)	99.0 (97.9, 99.5)	0.825 (0.738, 0.911)
HPV	97.0 (82.5, 99.8)	80.6 (77.8, 83.2)	16.2 (11.5, 22.3)	99.9 (99.1, 100.0)	0.888 (0.847, 0.928)
OCT	81.8 (63.9, 92.4)	87.4 (84.9, 89.5)	20.1 (13.9, 28.1)	99.2 (98.2, 99.7)	0.846 (0.769, 0.923)
HPV untyped + cytology	72.7 (54.2, 86.1)	91.3 (89.1, 93.1)	24.5 (16.6, 83.4)	98.9 (97.8, 99.4)	0.820 (0.730, 0.910)
HPV untyped +OCT	81.8 (63.9, 92.4)	93.8 (91.9, 95.3)	33.8 (23.8, 45.3)	99.3 (98.3, 99.7)	0.878 (0.800, 0.955)
HPV typing + cytology*	93.9 (78.4, 98.9)	88.4 (86.0, 90.4)	23.8 (17.0, 32.3)	99.7 (98.9, 100.0)	0.911 (0.862, 0.960)
HPV typing+OCT*	93.9 (78.4, 98.9)	90.0 (87.7, 91.9)	26.7 (19.1, 35.9)	99.7 (99.0, 100.0)	0.920 (0.871, 0.968)

Cytology is considered positive with ASC-US+. *Typing plus OCT or cytology means sub-type detection for HPV-positive women, direct referral for colposcopy for HPV 16/18 positive women, referral for OCT or cytology for other HPV types positive women, colposcopy for OCT positive or abnormal cytology.

PPV, positive predictive value; NPV, negative predictive value; AUC, area under the curve; CIN2+, cervical intraepithelial neoplasia grade 2 or higher; HPV, human papillomavirus; OCT, optical coherence tomography; CIN3+, cervical intraepithelial neoplasia grade 3 or higher.

The clinical accuracy analysis of triage among HPV-positive women by different methods is shown in **Table 2**. When using CIN2+ as the endpoint, the sensitivity of HPV typing plus OCT triage or cytological triage was higher (≥ 85%) than that of HPV untyped plus cytological triage (91.7% vs. 70.0%, *P*=0.002; 85.0% vs. 70.0%, *P*=0.004). The sensitivity of HPV typing combined with OCT triage was higher than that of HPV untyping plus OCT triage (91.7% vs. 80.0%, *P*=0.016). The specificity of HPV typing plus OCT triage was higher than that of HPV typing plus cytological triage (92.6% vs. 90.4%, *P*=0.005). OCT triage after HPV typing was the strategy with the highest AUC (0.921).

Accuracy analysis indicated that HPV typing plus OCT triage in HPV-positive women or HPV typing plus cytological triage had higher sensitivity for detecting CIN3+ (>90%). The sensitivity of HPV typing plus OCT triage and HPV typing plus cytological triage was higher than that of HPV untyped cytological triage (93.9% vs. 72.7%, *P*=0.039). The specificity of HPV untyped OCT triage was higher than that of HPV untyped cytological triage (93.8% vs. 91.3%, *P*=0.013). OCT triage after HPV typing had a higher AUC (0.920).

## Discussion

This study aimed to validate the potential of OCT as a new technology for the early diagnosis of cervical cancer and precancerous lesions and assess its utility in the triage of HPV-positive patients. Regardless of considering CIN2+ or CIN3+ as the clinical endpoint, the detection rate was higher among OCT-positive women than among cytological ASC-US+ women. Furthermore, among HPV-positive women, the detection rate of CIN2+ was lower among OCT-negative women compared to NILM. In the triage analysis of HPV-positive women, the sensitivity and specificity of HPV typing combined with OCT were superior to those of HPV typing combined with cytology, indicating the promising role of HPV typing combined with OCT in the triage of HPV-positive women.

It usually takes 8 to 10 years for precancerous lesions to progress into cervical cancer, and early screening may help the detection of early-stage cancer and reduce the risk of death, which is important for the prevention and treatment of cervical cancer (10). Numerous studies have confirmed that HPV testing is an accurate method of initial screening for cervical cancer (11–13). However, HPV infection is a transient infection and most women will be negative for HPV virus on their own after a short while (14). With the increased use of HPV tests for the primary screening of cervical cancer, many women with HPV infection have been identified in recent years. Colposcopy or biopsy will increase the psychological burden of these women and waste medical resources. Therefore, it is critical to provide the necessary triage for HPV-positive women. Currently, the combination of HPV test and cytological assessment is commonly used for initial screening. However, cytological diagnosis relies on the experience of cytopathologists, which is different in different regions and difficult to improve in the short term. Therefore, optical coherence chromatography a real-time, non-invasive, and accurate method, can be considered for the early diagnosis of cervical cancer.

OCT examination is a three-dimensional imaging technology developed in the 1990s (15). It uses the principle of weakly coherent light interference to image superficial tissues by detecting the echo time delay and echo intensity of back-scattered and back-reflected waves. After detecting and processing the backscattered light echo signal, the device forms three-dimensional images of the internal structure of the tissue. Different tissues respond to optical signals differently. The longitudinal resolution can generally reach 1-10 microns, and the horizontal resolution can also reach microns (16). OCT is a real-time, high-resolution, non-invasive imaging tool (6) that can present the internal structure of superficial tissues. It has been used in ophthalmology (17), cardiology (18), stomatology, and other areas (19). Currently, few reports are available on the use of OCT for diagnosing precancerous lesions of cervical cancer. OCT can be combined with HPV as an alternative for cytology. The combination is an optimal method for triage in HPV-positive women.

In this study, we assessed the accuracy of OCT as a primary screening method and combined screening with HPV test and HPV-positive female triage technique for the early diagnosis of cervical cancer and precancerous lesions. Of 883 enrolled women, 731 women were classified as normal, 92 women were classified as CIN1, 27 women were classified as CIN2, and 33 women were classified as CIN3 or more. Consistent with previous studies, our results showed that as a primary screening method for diagnosing high-grade cervical lesions, the sensitivity of OCT was 83.3% and the specificity was 89.8% (6, 20). These findings indicated that OCT has good screening efficacy for the diagnosis of high-grade cervical lesions and high clinical value. HPV is the primary cause of cervical dysplasia and cervical cancer, with persistent HPV infection considered a major risk factor for the development and recurrence of cervical lesions (21). Bogani et al. (22) demonstrated recurrence rates of 7.46% and 13.1% at 6 and 12 months after the initial conization, respectively. Hence, integrating the results of HPV test is crucial to assess the diagnostic performance of OCT. Among HPV-positive women and HPV16/18-positive women, the detection rates of CIN2+ and CIN3+ were significantly lower among OCT-negative women than among those with no abnormalities in cytological assessments, suggesting the greater reliability of OCT. In terms of sensitivity, specificity, and AUC, HPV combined with OCT was superior to HPV test combined with cytological assessment for the diagnosis of CIN3+. Therefore, HPV combined with OCT has a good clinical efficacy for the diagnosis of cervical lesions and can serve as an alternative method in areas where cytologists are scarce. In addition, OCT is simple and can be applied after simple training, requiring no special laboratory techniques or personnel. It can also report the results in a real-time manner during the gynecologic examination and determine whether colposcopy is needed. OCT can reduce unnecessary biopsies and decrease the need for secondary recalls.

Finally, it is important to mention the limitations of this study. Firstly, the number of HPV-positive cases was relatively small in this study; thus, the results might be biased. Additionally, the results of OCT in this study were limited to negative and positive categories, which is simplistic compared to the results of cytology. Furthermore, we analyzed data from only two sources, which raises concerns about the generalizability of our findings. Future large-scale, multicenter clinical studies are needed to validate the value of OCT in the early diagnosis of cervical cancer and precancerous lesions and assess its value in the triage of HPV-positive women. These efforts can help overcome the limitations of this study and provide more robust evidence for the use of OCT.

## Conclusion

In conclusion, OCT is a non-invasive, real-time, rapid, and high-resolution imaging technique with high accuracy for the early diagnosis of cervical cancer and precancerous lesions.

## Data availability statement

The original contributions presented in the study are included in the article/supplementary material. Further inquiries can be directed to the corresponding authors.

## Ethics statement

The studies involving humans were approved by Ethics Committee of Liaoning Cancer Hospital (Approval number: 20200101). The studies were conducted in accordance with the local legislation and institutional requirements. The participants provided their written informed consent to participate in this study. Written informed consent was obtained from the individual(s) for the publication of any potentially identifiable images or data included in this article.

## Author contributions

XC: Writing – review & editing, Writing – original draft, Supervision, Formal analysis, Data curation. DY: Writing – review & editing, Project administration, Data curation. JZ: Writing – review & editing, Project administration, Data curation. YZ: Writing – review & editing, Formal analysis. ZC: Writing – review & editing, Project administration. CW: Writing – review & editing, Supervision. YQ: Writing – review & editing, Supervision.
